# Assessment of the Ecological Protection Effectiveness of Protected Areas Using Propensity Score Matching: A Case Study in Sichuan, China

**DOI:** 10.3390/ijerph19084920

**Published:** 2022-04-18

**Authors:** Zhifeng Zhang, Yuping Tang, Hongyi Pan, Caiyi Yao, Tianyi Zhang

**Affiliations:** 1 Key Laboratory of Land Resources Evaluation and Monitoring in Southwest, Ministry of Education, Chengdu 610068, China; bubble99@stu.sicnu.edu.cn (Z.Z.); 20201103008@stu.sicnu.edu.cn (Y.T.); 20211101030@stu.sicnu.edu.cn (C.Y.); 20211101031@stu.sicnu.edu.cn (T.Z.); 2 The Faculty of Geography and Resources Sciences, Sichuan Normal University, Chengdu 610068, China

**Keywords:** protected areas, NPP, PSM, ecological protection effectiveness, Sichuan Province

## Abstract

Protected areas constitute a global strategic resource for enhancing the effectiveness of ecological protection, which can alleviate the impact of unsustainable human production and living activities on the ecological environment. However, the spatiotemporal evolution of ecological protection effectiveness needs to be quantitatively revealed. The net primary productivity (NPP) of plants is an important measure of the effectiveness of ecological protection efforts. The main purpose of this study is to use the relative change in the annual average NPP to evaluate the ecological protection effectiveness of protected areas. We compared the historical changes in the annual average NPP of protected areas in Sichuan Province from 2000 to 2019. We added the spatial coordinates to the impact factor system and adopted propensity score matching (PSM) in a quasi-natural experimental method to determine the experimental group and the control group. The ecological protection effectiveness of the protected areas in the study area in 2000, 2005, 2010, 2015, and 2019 was measured and classified into three types of changes in protection effectiveness, namely effective, ineffective, or fluctuating. According to the administrative level, type, and spatial distribution, we determined the number and type of changes in the protection effectiveness of different protected areas. The results show that the annual average NPP of the protected areas in Sichuan Province generally fluctuated. The annual average NPP increased in 95.47% of the total protected area and decreased in 4.53%. The overall protection effectiveness of protected areas was positive and significant and gradually improved. Effective protected areas at the national, provincial, and county levels accounted for 40.27% of the total number of protected areas, and the other 14.77% of effective protected area was managed at other administrative levels. Among the different types of protected areas, the proportion of effective protected areas was highest in wild animal protected areas, followed by forest ecology protected areas, wild plant protected areas, and wetland ecology protected areas. The results of this study can provide an important reference for the verification and improvement of the ecological protection effectiveness of various protected areas.

## 1. Introduction

Since the establishment of the first protected area in the United States in 1872, the scale and number of protected areas in the world have grown. In 2020, the Protected Planet Report 2020 issued by the United Nations Environment Program and the International Union for Conservation of Nature pointed out that the area of terrestrial and inland water ecosystems reached 2250 × 10^4^ square kilometers, of which areas recorded as terrestrial protected areas and reservations accounted for 16.64%. Past studies have shown that the establishment of protected areas is of great importance to the maintenance of biodiversity and ecological security [1,2] and even plays a key role in the protection of cultural landscapes [3,4]. However, the actual ecological protection effectiveness of protected areas is disturbed by various human activities. For example, different land use practices change the vegetation coverage of the land, thereby affecting the effectiveness of ecological protection [5].

In the context of urbanization and rapid economic growth, to protect ecosystems such as forests and wetlands [6], China has established a large number of protected areas. As of the end of 2020, 2676 protected areas have been established in China, with a total area of 150 × 10^4^ square kilometers, including 474 national protected areas with a combined area of 98 × 10^4^ square kilometers (data from the Ministry of Natural Resources of the People’s Republic of China). The number of protected areas in China is constantly growing, and in the rapidly developing process of the management and construction of protected areas, the issue of ecological protection effectiveness of protected areas has attracted gradually increasing attention from scholars [7,8].

Net primary productivity (NPP) refers to the amount of carbon captured by plants via photosynthesis in one year minus the amount of carbon required for plant respiration, which reflects the productivity and quality of the ecosystem. Many studies have found that NPP is closely related to species richness and population density [9]. Thus, NPP provides valuable information on the characteristics of ecosystem change. The more stable and powerful an ecosystem is, the greater the NPP [10]. Various measures taken by humans to protect the environment can positively affect NPP [11]. Remote sensing technology can provide long-term monitoring images of ground objects, making the acquisition of NPP estimates relatively simple [12]. As a simple instrumental variable, NPP facilitates the study of large-scale areas. Therefore, NPP can be used to assess the ecological protection effectiveness of protected areas [13].

Studies on the ecological protection effectiveness of protected areas have evaluated the protection effectiveness of single protected areas [14] or certain types of protected areas [15] or performed horizontal comparisons of the protection effectiveness of protected areas [16]. However, few studies have used remote sensing images to evaluate the ecological protection effectiveness of all protected areas within a specified region. In terms of methods for researching the ecological protection effectiveness of protected areas, studies generally make direct longitudinal comparisons of the ecological protection effectiveness before and after the establishment of protected areas [17]. This method does not consider the external impacts of protected areas or compare them with nonprotected areas, so it is difficult to draw objective conclusions. In addition, some scholars have compared the internal states of protected areas with conditions external to those areas [18]. However, this method cannot overcome the problem of sample selection; it is impossible to avoid the impacts of factors such as temperature, precipitation, and traffic accessibility of the protected areas. The conclusions drawn based on this method may not be consistent with the actual situation. Therefore, it is necessary to quantitatively study the impacts of the establishment and management of protected areas, as well as external factors, on ecosystems. To address the difficulties in selecting samples within and outside protected areas, some scholars have introduced the sample matching method [19,20]. Propensity score matching (PSM) was first proposed by Rosenbaum and Rubin [21]. The core idea of PSM is to calculate the propensity score of a sample based on variables and to use the propensity scores of the experimental group and the control group as the functional distance for matching so that the observed variables are as similar as possible, thereby solving the problem of bias in sample selection. The PSM method has been widely used in medicine [22,23] and public health [24,25,26]. Compared with direct comparisons between conditions internal and external to protected areas, the PSM-based quasi-natural experimental model is more scientific and reliable [27]. However, in existing studies using the PSM-based quasi-natural experimental model, spatial factors were not considered when selecting factors, which affects the accuracy of the matching results.

Sichuan has always attached importance to the protection of the ecological environment and is one of the provinces with the greatest number and area of protected areas in China (data from the Ministry of Natural Resources of the People’s Republic of China). To promote the management of protected areas, Sichuan Province has successively promulgated corresponding regulations governing protected areas. In this paper, NPP was used as an indicator to evaluate the ecological protection effectiveness of protected areas in Sichuan Province from 2000 to 2019, and the PSM method was used to eliminate bias in the selection of samples within and outside of protected areas. In this paper, we examine (1) changes in the spatiotemporal patterns of the NPP of protected areas between 2000 and 2019 and (2) the distribution of the ecological protection effectiveness of protected areas among administrative levels and protected area types.

## 2. Materials and Methods

### 2.1. Boundaries of Protected Areas

**Figure 1:**
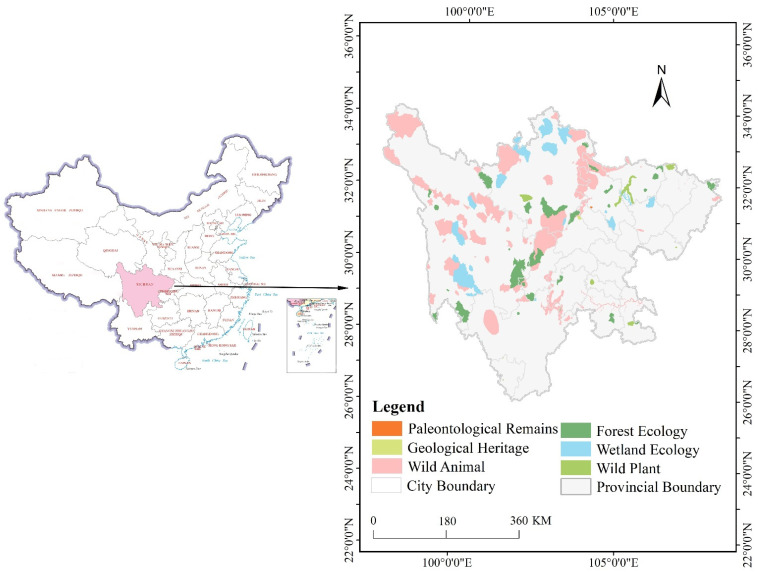
Types of protected areas.

The protected areas in this study refer to the various nature reserves under the nature reserve system in China. The protected areas are classified into nine types, including forest ecology, paleontological remains, geological heritage, etc. The protected areas are also categorized into national, provincial, county, and other administrative levels [28]. The data came from the specimen resource sharing platform of the China Nature Reserve (http://www.papc.cn/, accessed on 15 October 2021) and were integrated with data on the ecological functional areas of Chinese nature reserves in ArcGIS Online (http://120.26.232.88:6080/arcgis/rest/services/ROOT/HJMGQ/MapServer, accessed on 17 October 2021). We combined these data with the latest Giant Panda National Park System data to obtain the final boundary, including a total of 149 protected areas (Figure 1). The protected areas (97°21′ E to 108°31′ E longitude, 26°03′ N to 34°19′ N latitude) are situated in Sichuan, China, including six types of protected areas, namely paleontological remains, geological heritage, forest ecology, wetland ecology, wild animal, and wild plant, representing considerable diversity. The protected areas in Sichuan include 40 national, 40 provincial, 44 county-level, and many other protected areas.

### 2.2. Net Primary Productivity (NPP)

The annual NPP product data for 2000–2019 came from the United States Geological Survey (USGS) (https://e4ftl01.cr.usgs.gov/MOLT/MOD17A3HGF.006/, accessed on 13 October 2021). The data layer is the sum of all eight-day net photosynthetic products with a resolution of 500 m. In this study, the final NPP dataset was obtained through tools such as “Define Projection” and “Extract by Mask”.

### 2.3. Propensity Score Matching Data

The road data were sourced from the National Earth System Science Data Center (http://www.geodata.cn, accessed on 14 October 2021). The highways, railways, and provincial roads in the study area were extracted and merged into a layer of main roads, and we calculated the Euclidean distance to characterize traffic accessibility. Elevation, slope, precipitation, average temperature, and land use type were obtained from the Resource and Environmental Science and Data Center of the Chinese Academy of Sciences (https://www.resdc.cn/, accessed on 14 October 2021). The annual precipitation and annual average temperature were obtained by spatial interpolation. The spatial coordinates of the experimental and control groups were extracted from ArcGIS.

### 2.4. Trend Analysis Method

Using pixels as the basic unit, the interannual NPP trends of the protected areas were studied using univariate linear regression analysis to reflect the trends and characteristics of the NPP of the vegetation in different years [29,30]. The formula for calculating the degree of change in NPP is as follows:

(1) $$K=\frac{n\times \sum_{i=1}^{n}\left(i\times NPP_{i}\right)-\sum_{i=1}^{n}i\sum_{i=1}^{n}NPP_{i}}{n\times \sum_{i=1}^{n}i^{2}-{\left(\sum_{i=1}^{n}i\right)}^{2}}$$

where K is the slope of the multiyear regression of a single pixel; n is the number of years; and $NPP_{i}$ represents the value of a certain point in the ith year, where i = 1, 2, 3, 4, 5 …, 20. When *K* > 0, NPP increases with time; when K < 0, NPP declines with time. The slope, K, is used to characterize the degree of change in NPP over a total of 20 years from 2000 to 2019 [30].

(2) $$NPP_{change}=\frac{K}{NPP_{mean}}\times n\times 100\%$$

$NPP_{change}$ is expressed as a percentage, $NPP_{mean}$ represents the average NPP for a total of 20 years from 2000 to 2019, and *n* is the number of years.

In ArcGIS, the average annual NPP value of a single protected area was obtained using the “Zonal Statistics” function. After the data were derived, the annual average NPP and the 20-year average NPP of the protected areas in Sichuan Province were calculated, and the spatiotemporal patterns of NPP change were analyzed.

### 2.5. Propensity Score Matching (PSM)

PSM refers to the estimation of the probabilities of occurrence of a sample point, i, in the experimental group and the control group based on a certain influencing factor, $M_{i}$. Based on the propensity scores of the experimental group and the control group, nearest-neighbor matching, nuclear matching, and other rules were used for matching [31]. The points in the experimental group and the control group that were closest to each other under multiple factors were selected to eliminate sample-selection bias [32].

(3) $$p\left(M_{i}\right)=pr\left(PA_{i}=1\left|M_{i}\right.\right)=F\left(f\left(M_{i}\right)\right)$$

$p\left(M_{i}\right)$ is the propensity score of the sample point i, $PA_{i}=0$ for the control group, and $PA_{i}$ = 1 for the experimental group. $f\left(M_{i}\right)$ is a linear function, whereas $F\left(x\right)$ is a logical function.

**Figure 2:**
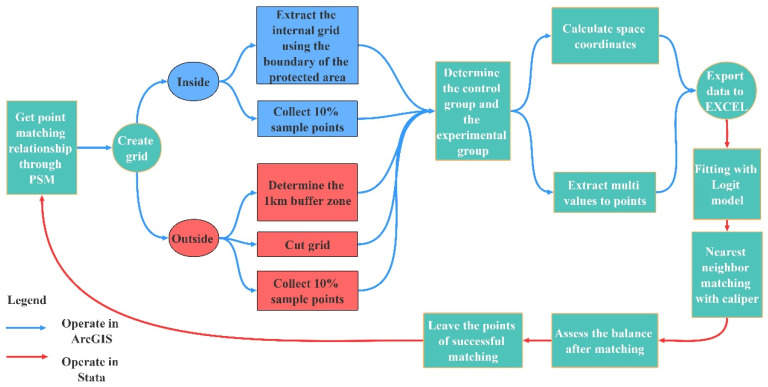
PSM technical route.

Based on an existing study [33,34,35,36], seven factors, including elevation, slope, annual average precipitation, annual average temperature, distance to a main road, distance to urban construction land, and land use type, were selected. These factors impact the NPP of the protected areas. In addition, the spatial coordinates were considered to improve the accuracy of internal and external point matching. In this study, a grid of the same size was established in the study area based on NPP with a resolution of 500 m. The grid was extracted from the boundaries of protected areas, and 10% of the sample points in the protected areas were selected as the experimental group through the ArcGIS “Subset” tool. The minimum distances between the protected areas in the study area are mostly more than 1 km. To avoid the impact of the spillover effect [37], 10% of the grid’s center points 1 km outside the protected areas were selected as the sample points of the control group after multiple experiments. The seven factors in 2000 and the corresponding spatial coordinates were matched to points, and the logit method was used to estimate the probabilities of the experimental group and the control group. The matching relationship between the experimental group within the protected areas and the control group outside the protected areas was obtained according to nearest-neighbor matching with caliper (Figure 2).

### 2.6. Protection Effectiveness Index

The difference between the mean NPP in a certain protected area and the mean NPP of the matched control group was used to calculate the ecological protection effectiveness of that protected area.

(4) $$Pe=\frac{\sum_{i=1}^{n_{1}}\Delta pixel_{i}}{n_{1}}-\frac{\sum_{j=1}^{n_{0}}\Delta point_{j}}{n_{0}}$$

Pe refers to the ecological protection effectiveness of a certain protected area; $\Delta pixel_{i}$ is the sum of the NPP of pixel, i, in a protected area in 2000, 2005, 2010, 2015, and 2019; $n_{1}$ is the total number of protected areas in Sichuan Province; $\Delta point_{j}$ is the sum of the NPP of the sample point, j, of the control group in 2000, 2005, 2010, 2015, and 2019 after matching; and $n_{0}$ is the number of sample points in the control group after matching. If Pe is greater than 0, then NPP inside the protected area is high, and the ecological protection effectiveness is positive. If Pe is less than 0, the ecological protection effectiveness of the protected area is negative.

## 3. Results

### 3.1. Analysis of the Spatiotemporal Patterns of NPP Change in Protected Areas

**Figure 3:**
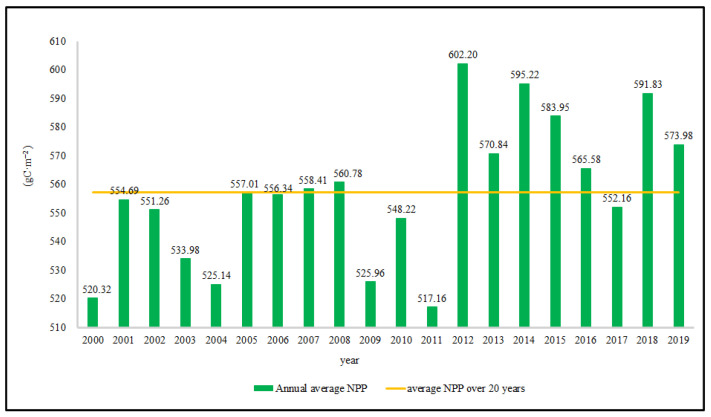
Annual and overall average NPP in protected areas of Sichuan Province (2000–2019).

Figure 3 shows that the annual average NPP of the study area fluctuated between 517 and 603 gC·m^−2^, increasing overall. Based on the annual average NPP, the average NPP over the 20-year period (2000–2019) was 557.25 gC·m^−2^. Before 2011, the annual average NPP was relatively low, and overall, it improved over time. During the study period, the annual average NPP was as low as 517.16 gC·m^−2^ in 2011, which is 7.75% lower than the average; in 2012, it was as high as 602.20 gC·m^−2^, which is 16.44% higher than the average.

**Figure 4:**
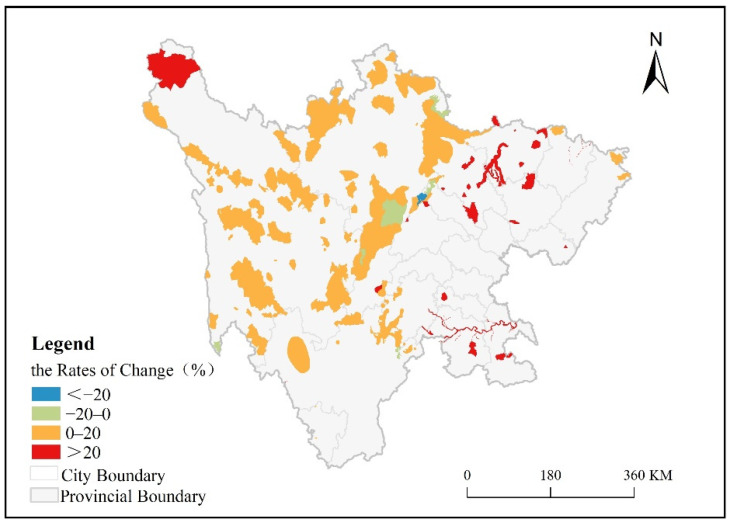
Rates of change in annual average NPP in Sichuan protected areas over 20 years.

The rates of change in the annual average NPP of the protected areas in the study over the 20-year period (2000–2019) were calculated using Equations (1) and (2) (Figure 4). The changes in the study period were classified by four types of trends, namely significant decrease (<−20%), slight decrease (−20% to 0%), stable increase (0% to 20%), and significant increase (>20%). The results showed that between 2000 and 2019, the annual average NPP increased overall in the study area and declined in only a few areas in the western part of the study area. The protected areas with increasing annual average NPP trends accounted for 95.47% of the total area, and the protected areas with decreasing annual average NPP trends accounted for 4.53% of the total area.

In the protected areas of Sichuan Province, one protected area showed a significant decrease in the annual average NPP, and this protected area accounted for 0.33% of the total area of protected areas. Eight protected areas showed slight decreases in annual average NPP, and they were scattered in the western and northern parts of the study area, accounting for 4.20% of the total protected area. The number of protected areas with stable increases in the annual average NPP was the largest, totaling 113, and these protected areas were mainly distributed in the western part of the study area, accounting for 81.94% of the total protected area. A total of 27 protected areas showed significant increases in the annual average NPP, and they were mainly distributed in the northern, southern, and northwestern portions of the study area, accounting for 13.52% of the total protected area.

### 3.2. Analysis of Protective Effectiveness of Protected Areas

The operation followed the technical route shown in Figure 2. Ultimately, the experimental group retained 28,262 points, and the control group retained 63,936 points (Figures S1 and S2). We verified the matching accuracy by means of a balance test and a common support test. The results demonstrated that the matching accuracy is relatively high (see Figure S3 and Table S1 for more details).

**Figure 5:**
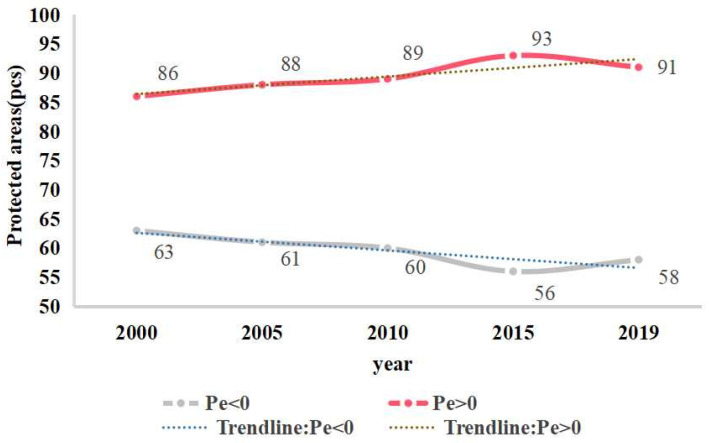
Statistics of changes in protection effectiveness.

The ecological protection effectiveness of the protected areas in Sichuan, China, in 2000, 2005, 2010, 2015, and 2019 was obtained based on Equation (4) (Figure 5). The results showed that from 2000 to 2015, the number of protected areas with Pe > 0 increased slightly, and the proportion of protected areas with Pe > 0 gradually increased. The ecological protection effectiveness was most prominent in 2015, and the number of protected areas with Pe > 0 was as high as 93 in 2015, an increase of nearly 8.14% compared with that in 2000. After 2015, the ecological protection effectiveness weakened slightly. However, the number of protected areas with Pe < 0 decreased overall. From 2000 to 2019, the trendline of protected areas with Pe > 0 in Sichuan Province showed an increasing trend.

**Figure 6:**
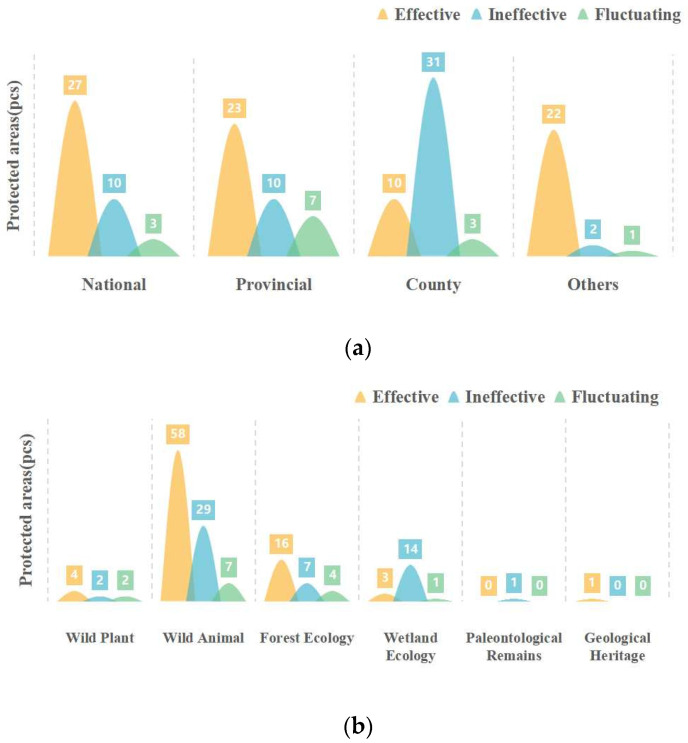
(**a**) Statistics by administrative level. (**b**) Statistics by type.

Based on the calculated Pe results, the changes in the ecological protection effectiveness of the protected areas in Sichuan Province from 2000 to 2019 were classified into three types: effective, ineffective, and fluctuating. Ecological protection was considered effective if Pe was >0 in all five years (2000, 2005, 2010, 2015, and 2019) and the ecological protection effectiveness of the protected areas was thus consistently effective. If Pe was <0 in all five years, then the ecological protection effectiveness was considered ineffective. If Pe was positive in some years and negative in others, then the ecological protection effectiveness was considered to be fluctuating (for the specific list, please refer to Table S2 of Supplementary Materials). First, statistical analysis was performed based on the administrative level of each protected area (Figure 6a). A large proportion of the national and provincial protected areas were effective from 2000 to 2019. A total of 50 national and provincial protected areas exhibited Pe > 0 in all five years (2000, 2005, 2010, 2015, and 2019), indicating that these protected areas played a positive role in promoting the protection of the regional ecological environment. However, 10 national and 10 provincial protected areas were consistently ineffective. Compared with county-level protected areas, a high proportion of the protected areas of other (i.e., other than national, provincial, or county) administrative levels were consistently effective. This phenomenon occurred because most of the protected areas are relatively remote, their surrounding areas are sparsely populated, and their vegetation is less damaged. The number of ineffective county-level protected areas (31) was greatest, accounting for approximately 20.81% of the total number of protected areas (149). The overall number of fluctuating protected areas was small, and they were mostly provincial protected areas.

The classification of protected areas by effectiveness was also examined for each type of protected area (Figure 6b). Wild animal protected areas accounted for nearly 63.09% of all protected areas, and the number of effective types of ecological protection effectiveness was 58, which is higher than the numbers for other types of protected areas. The number of forest ecology protected areas ranked second, followed by the number of wild plant protected areas, the number of wetland ecology protected areas, and the number of geological heritage protected areas. Among the ineffective protected areas, wild animal protected areas and wetland ecology protected areas accounted for a high proportion, totaling 43 protected areas, and the forest ecology protected areas accounted for only 4.70%. The only paleontological remains protected area (Suishui Sponge Reef Protected Area) was consistently ineffective, which is closely related to its low vegetation coverage and low NPP. The fluctuating protected areas were also mostly wild animal protected areas, and the overall proportion of fluctuating protected areas was small.

**Figure 7:**
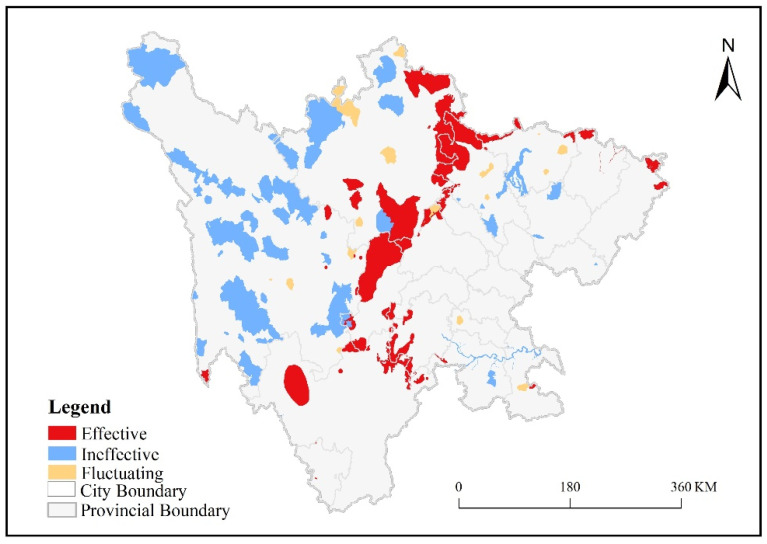
Types of variation in protection effectiveness.

To enhance the contrast of spatial differences, the spatial locations of the types of variation in ecological protection effectiveness of 149 protected areas are presented in Figure 7.

Figure 7 shows that between 2000 and 2019, the effective protected areas were mainly located in the marginal mountains of the Sichuan Basin and were mainly distributed in the northern portion of the study area. Among the 105 protected areas in the western part of the study area, more than half behaved as consistently effective between 2000 and 2019. Most of the ineffective protected areas were located in the western part of the study area, and their spatial distribution was relatively concentrated. Seven ineffective protected areas were concentrated in the northern part of the study area, accounting for 4.70% of the total number of protected areas, whereas a small number of ineffective protected areas was scattered in the southern and eastern parts of the study area. Only a small number of fluctuating protected areas was found in the study area, and they were scattered within the study area but were more concentrated in the western alpine plateau area.

## 4. Discussion

In this study, we used NPP as an instrumental variable to characterize the ecological protection effectiveness of protected areas in Sichuan Province. Figure 3 shows that from 2000 to 2019, the annual average NPP of protected areas in Sichuan showed fluctuating growth. Before 2011, the annual average NPP was lower than the overall average NPP, and after 2011, the annual average NPP grew rapidly and was higher than the overall average NPP. This may be because the government did not strictly restrict human activities after the implementation of the protected area policy in 2000. Coupled with people’s lack of awareness of protected areas, the ecological environment of protected areas was negatively affected to some extent [38]. In 2009, the government revised the management regulations of nature reserves to improve the protection of nature reserves. However, due to the lag in the policy implementation process, the ecological environment was not significantly improved until the latter period [39]. We observed that from 2000 to 2019, the trend of average NPP change in protected areas in Sichuan Province showed that NPP growth occurred in 95.47% of the total area of protected areas and that the ecological environment was improved. However, because temperature and precipitation also affect NPP [40], the changes cannot be proven to influenced only by protected area policy. Therefore, in this study, we controlled these interfering factors to minimize the sample selection bias via PSM, and the ecological protection effectiveness of protected areas was analyzed in depth. Through the improved-propensity score-matching method, the ecological protection effectiveness of each protected area was calculated according to Equation (4), and the results showed that within 20 years, the overall ecological protection effectiveness of the protected areas in Sichuan Province increased (Figure 5). Then, we conducted an in-depth study on the ecological protection effectiveness of protected areas from three perspectives: level, type, and spatial location.

After statistical analysis of changes in the effectiveness of protected areas according to different levels of standards, we found that many national and provincial protected areas consistently and effectively maintain an internal ecological balance (Figure 6a), which is consistent with the conclusions of a previous study [41]. However, among the ineffective protected areas, national and provincial protected areas were consistently ineffective in 2000, 2005, 2010, 2015, and 2019. County-level protected areas accounted for the greatest proportion of ineffective protected areas. Ineffective protected areas were mainly concentrated in the western alpine plateau area, where the ecological environment is relatively fragile [42]. The local governments in these areas may not have sufficient backup support for the implementation of policies due to their low economic strength, which has hindered effective improvement of the ecological environment [39]. In the future, we should first ensure that all national protected areas can truly achieve continuous and effective protection. Taking advantage of the short distances between these protected areas, we merged and reorganized them with scattered county-level and other levels of protected areas to form a networked and systematic system of protected areas [43]. A good example of this practice is the current national park system implemented in China [44]. This practice should be the main method for constructing a framework for improving the ecological protection effectiveness of areas protected at different administrative levels.

From the perspective of different types of protected areas, most of the effective wild animal protected areas in the study area are giant panda (*Ailurus fulgens*) protected areas (Figure 6b). Giant pandas are national rare animals, and protecting their habitat has received increasing attention in recent years [45]. The government has invested a large amount of human, material, and financial resources to implement relevant projects and protection policies to improve the habitat of giant pandas, so most giant panda protected areas are effective [46]. However, the ecological protection effectiveness of some protected areas remains suboptimal. For instance, 27 wild animal protected areas located in the western alpine plateau area, such as Kaniang town, Gajin, Fozhu, and Suochong, are highly sensitive to the ecological environment. The fences that are built to delineate protected areas affect the ecological processes of their habitats to some extent, reducing the vegetation cover [47]. Simultaneously, ineffective protected areas may have resulted from the late establishment of protection, lags in policies, or inadequate management and control systems [48]. Economic activities to improve livelihoods have generated disturbances in wetland and forest ecology protected areas, affecting the ecological environments of these protected areas [49]. In the process of developing areas protecting paleontological remains, tourism resources, infrastructure construction, and unauthorized visits by humans cause a certain negative impact on the ecological environments of these areas [50,51]. To enhance the diversification of ecological protection, different types of protected areas should be coordinated in the implementation of policies. In addition, the government needs to develop alternative livelihoods to reduce the possibility of human activities affecting wetland and forest ecology in the study area [52]. The development of paleontological remains or geological heritage should be reasonably controlled, and environmental management should be improved.

From the perspective of spatial distribution, the number of effective protected areas in the Longmen Mountain and Xiaoliang Mountain areas accounts for almost half of the total number of existing protected areas (Figure 7). The protected areas in this region are close to major cities and are advantageous in terms of the implementation of ecological and environmental protection policies and economic investment. However, the ineffective protected areas were mostly concentrated in the western part of the study area because a large number of protected areas are distributed in the western part of the study area, and most of them are in alpine plateaus, with relatively low NPP and high ecological sensitivity [53,54]. In addition, the level of economic development in the region is low, the ability of local governments to control relevant factors is weak, and policy measures have not been effectively implemented [53]. In the eastern part of the study area, the ecological protection of wetlands (mainly four wetland protected areas, including the Wenxixi River, Gouxihe Wetland, Mianyang Egret Forest Protected Area, and Luhu White Stork Grey Crane Protected Area) was consistently ineffective and may also be limited by the low level local economic development [55]. Furthermore, the large area of water bodies in the wetland ecology protected areas affected the ability of NPP to characterize the ecological protection effectiveness of these protected areas. Therefore, in the future, financial investment should be strengthened [56], and high-intensity human activities, such as overgrazing, should be restricted in the protected areas in the remote mountainous areas of western China [57]. In the ineffective wetland ecology protected areas in the eastern part of the study area, the rivers and lakes should be used as the core, and artificial disturbance measures should be used to promote the restoration of natural vegetation. Alternatively, new vegetation can be established to restore the vegetation communities surrounding water bodies [58].

At present, few studies have investigated the ecological protection effectiveness of multitype or multilevel protected areas [59,60], and most of these studies have focused on the analysis of single-type or single-level protected areas [61,62,63], so they cannot achieve a multiangle assessment of the overall ecological protection effectiveness of protected areas. Additionally, to address the problem of matching points within and outside of the protected area by PSM, some scholars have introduced PSM in a quasi-natural experimental model to compare the differences in the human disturbance index within and outside of protected areas to reflect the effectiveness of the protected areas [41]. However, these studies failed to take into account that when matching points, a problem may arise in which the internal and external matching points are far apart in space. Therefore, we not only considered the main influencing factors, such as traffic accessibility and differences in natural conditions, but also introduced spatial coordinates so that we were able to improve the matching accuracy by selecting the control group with short spatial distances from protected points. After 2000, China began to implement a policy of conversion of cropland to forest and the natural forest protection project [64]. Moreover, most of the protected areas were established during this period. Accordingly, this study used 2000 as the base year for point matching to better present the changes in ecological protection effectiveness after the implementation of these policies. The average NPP was then used to measure the ecological protection effectiveness from 2000 to 2019, and the types of overall changes in ecological protection effectiveness were determined based on the Pe values in 2000, 2005, 2010, 2015, and 2019. In this way, the spatiotemporal patterns of changes in the ecological protection effectiveness of the protected areas in Sichuan Province are shown at different administrative levels, for different protected area types, and for different spatial distributions.

It should be pointed out that due to the influence of multiple factors, the causes and mechanisms of the failure of protected areas of different levels, types, and spatial distributions to reach optimal ecological protection effectiveness still need to be further studied. In addition, from the perspective of the instrumental variables used in the study, the variables that characterize the ecological environment should not be limited to NPP alone. We can consider combining multiple indicators, such as the normalized difference vegetation index (NDVI) and leaf area index (LAI), to improve the comprehensiveness and scientific nature of the representation of the ecological environment. Moreover, the modal and maximum NPP for the control group and the experimental group can be used as indicators of ecological protection for further analysis to enhance understanding of the spatiotemporal patterns of changes in ecological protection effectiveness.

## 5. Conclusions

Studying the ecological protection effectiveness of protected areas is critical for their future development and planning. In this study, spatial coordinates were included in the influencing factors to more accurately determine the matching relationship between the experimental group and the control group. The ecological protection effectiveness of the protected areas in Sichuan Province was evaluated by calculating the differences between the NPP of the experimental group and the control group. The conclusions are as follows:

The annual average NPP of protected areas in Sichuan Province showed a fluctuating increase in the overall period from 2000 to 2019. In terms of spatial distribution, the protected areas with annual average NPP growth over the past 20 years accounted for 95.47% of the area of all protected areas, and these protected areas were widely distributed. The annual average NPP decreased in 4.53% of the total protected area, and these protected areas were mainly distributed in the western part of the study area.

The overall ecological protection effectiveness of the protected areas in the study area increased from 2000 to 2019. In 2015, 93 protected areas exhibited Pe > 0, the most favorable ecological protection effectiveness. Among the protected areas in Sichuan Province, between 2000 and 2019, the national and provincial protected areas with effective changes in ecological protection effectiveness accounted for 33.56% of the total, county-level protected areas accounted for 6.71%, and nearly 14.77% were other-level protected areas. Administrative-level protected areas displayed the greatest ecological protection effectiveness, indicating that the establishment or upgrading of administrative-level protected areas plays an important role in ecological protection. The proportion of effective protected areas was highest in wild animal protected areas, followed by forest ecology protected areas, wild plant protected areas, and wetland ecology protected areas. In terms of spatial distribution, the consistently effective protected areas were mainly distributed in the marginal mountains of the Sichuan Basin. The results show that the overall ecological protection effectiveness of the protected areas in Sichuan Province has been improving over the past 20 years. Studying the protection effectiveness of these protected areas can provide a reference for the construction of protected areas in the future and for research on other protected areas.

## Data Availability

Not applicable.

## Supplementary Materials

The following supporting information can be downloaded at https://www.mdpi.com/article/10.3390/ijerph19084920/s1: Figure S1: Points of the experimental group; Figure S2: Points of the control group; Figure S3: Nuclear density comparison chart; Table S1: Results of balance test; Table S2: List of the Types of Changes in Protection Effectiveness of Protected Area in Sichuan Province.

## References

[1] Ma B., Zhang Y.Q., Hou Y.L., Wen Y.L. (2020). Do Protected Areas Matter? A Systematic Review of the Social and Ecological Impacts of the Establishment of Protected Areas. Int. J. Environ. Res. Public Health.

[2] Eklund J., Blanchet F.G., Nyman J., Rocha R., Virtanen T., Cabeza M. (2016). Contrasting spatial and temporal trends of protected area effectiveness in mitigating deforestation in Madagascar. Biol. Conserv..

[3] Xu P., Wang Q., Jin J., Jin P. (2019). An increase in nighttime light detected for protected areas in mainland China based on VIIRS DNB data. Ecol. Indic..

[4] Yang H., Vina A., Winkler J.A., Chung M.G., Dou Y., Wang F., Zhang J., Tang Y., Connor T., Zhao Z. (2019). Effectiveness of China’s protected areas in reducing deforestation. Environ. Sci. Pollut. Res. Int..

[5] MacDougall A.S., McCann K.S., Gellner G., Turkington R. (2013). Diversity loss with persistent human disturbance increases vulnerability to ecosystem collapse. Nature.

[6] Ren G., Young S.S., Wang L., Wang W., Long Y., Wu R., Li J., Zhu J., Yu D.W. (2015). Effectiveness of China’s National Forest Protection Program and nature reserves. Conserv. Biol..

[7] Huang Y., Fu J., Wang W., Li J. (2019). Development of China’s nature reserves over the past 60 years: An overview. Land Use Policy.

[8] Rao Y., Zhang J., Wang K., Wu X. (2019). How to prioritize protected areas: A novel perspective using multidimensional land use characteristics. Land Use Policy.

[9] Luck G.W. (2007). The relationships between net primary productivity, human population density and species conservation. J. Biogeogr..

[10] Tilman D., Isbell F., Cowles J.M. (2014). Biodiversity and Ecosystem Functioning. Annu. Rev. Ecol. Evol. Syst..

[11] Teng M.J., Zeng L.X., Hu W.J., Wang P.C., Yan Z.G., He W., Zhang Y., Huang Z.L., Xiao W.F. (2020). The impacts of climate changes and human activities on net primary productivity vary across an ecotone zone in Northwest China. Sci. Total Environ..

[12] Powers R.P., Coops N.C., Nelson T., Wulder M.A. (2016). Evaluating Nature Reserve Design Efficacy in the Canadian Boreal Forest Using Time Series AVHRR Data. Can. J. Remote Sens..

[13] O’Neill D.W., Abson D.J. (2009). To settle or protect? A global analysis of net primary production in parks and urban areas. Ecol. Econ..

[14] Gu C.J., Zhao P., Chen Q., Li S.C., Li L.H., Liu L.S., Zhang Y.L. (2020). Forest Cover Change and the Effectiveness of Protected Areas in the Himalaya since 1998. Sustainability.

[15] Nord M., Ranlund A., Gustafsson L., Johansson V., Part T., Forslund P. (2019). The effectiveness of area protection to capture coastal bird richness and occurrence in the Swedish archipelago. Glob. Ecol. Conserv..

[16] Linkie M., Smith R.J., Leader-Williams N. (2004). Mapping and predicting deforestation patterns in the lowlands of Sumatra. Biodivers. Conserv..

[17] Gaveau D.L.A., Wandono H., Setiabudi F. (2007). Three decades of deforestation in southwest Sumatra: Have protected areas halted forest loss and logging, and promoted re-growth?. Biol. Conserv..

[18] Vačkář D., Harmáčková Z.V., Kaňková H., Stupková K. (2016). Human transformation of ecosystems: Comparing protected and unprotected areas with natural baselines. Ecol. Indic..

[19] Andam K.S., Ferraro P.J., Pfaff A., Sanchez-Azofeifa G.A., Robalino J.A. (2008). Measuring the effectiveness of protected area networks in reducing deforestation. Proc. Natl. Acad. Sci. USA.

[20] Mas J.F. (2005). Assessing protected area effectiveness using surrounding (buffer) areas environmentally similar to the target area. Environ. Monit. Assess..

[21] Rosenbaum P.R., Rubin D.B. (1983). The central role of the propensity score in observational studies for causal effects. Biometrika.

[22] Dang C., Wang M., Zhu F., Qin T., Qin R. (2021). Comparison of laparoscopic and open pancreaticoduodenectomy for the treatment of nonpancreatic periampullary adenocarcinomas: A propensity score matching analysis. Am. J. Surg..

[23] Oiwa K., Fujita K., Lee S., Morishita T., Tsukasaki H., Negoro E., Hara T., Tsurumi H., Ueda T., Yamauchi T. (2021). Prognostic impact of six versus eight cycles of standard regimen in patients with diffuse large B-cell lymphoma: Propensity score-matching analysis. ESMO Open.

[24] Benmarhnia T., Zhao X., Wang J., Macdonald M., Chen H. (2019). Evaluating the potential public health impacts of the Toronto cold weather program. Environ. Int..

[25] Singh A., Vellakkal S. (2021). Impact of public health programs on maternal and child health services and health outcomes in India: A systematic review. Soc. Sci. Med..

[26] Yanovitzky I., Zanutto E., Hornik R. (2005). Estimating causal effects of public health education campaigns using propensity score methodology. Eval. Program Plan..

[27] Coetzee B.W., Gaston K.J., Chown S.L. (2014). Local scale comparisons of biodiversity as a test for global protected area ecological performance: A meta-analysis. PLoS ONE.

[28] Duan W., Wen Y. (2017). Impacts of protected areas on local livelihoods: Evidence of giant panda biosphere reserves in Sichuan Province, China. Land Use Policy.

[29] Zhang Y., Gao J., Liu L., Wang Z., Ding M., Yang X. (2013). NDVI-based vegetation changes and their responses to climate change from 1982 to 2011: A case study in the Koshi River Basin in the middle Himalayas. Glob. Planet. Chang..

[30] Wu J., Gong Y., Wu J. (2018). Spatial distribution of nature reserves in China: Driving forces in the past and conservation challenges in the future. Land Use Policy.

[31] Abadie A., Imbens G.W. (2016). Matching on the Estimated Propensity Score. Econometrica.

[32] Olmus H., Bespinar E., Nazman E. (2019). Performance evaluation of some propensity score matching methods by using binary logistic regression model. Commun. Stat.-Simul. Comput..

[33] Oldekop J.A., Holmes G., Harris W.E., Evans K.L. (2016). A global assessment of the social and conservation outcomes of protected areas. Conserv. Biol..

[34] Zhang Y., Zhang C., Wang Z., Chen Y., Gang C., An R., Li J. (2016). Vegetation dynamics and its driving forces from climate change and human activities in the Three-River Source Region, China from 1982 to 2012. Sci. Total Environ..

[35] Chen T., Peng L., Liu S., Wang Q. (2017). Spatio-temporal pattern of net primary productivity in Hengduan Mountains area, China: Impacts of climate change and human activities. Chin. Geogr. Sci..

[36] Zhou Y., Yue D., Li C., Mu X., Guo J. (2021). Identifying the spatial drivers of net primary productivity: A case study in the Bailong River Basin, China. Glob. Ecol. Conserv..

[37] Bowker J.N., De Vos A., Ament J.M., Cumming G.S. (2017). Effectiveness of Africa’s tropical protected areas for maintaining forest cover. Conserv. Biol..

[38] Hernandez S., Benham C., Miller R.L., Sheaves M., Duce S. (2021). What drives modern protected area establishment in Australia?. Conserv. Sci. Pract..

[39] He M., Cliquet A. (2020). Challenges for Protected Areas Management in China. Sustainability.

[40] Bala G., Joshi J., Chaturvedi R.K., Gangamani H.V., Hashimoto H., Nemani R. (2013). Trends and Variability of AVHRR-Derived NPP in India. Remote Sens..

[41] Zhang H., Li X., Shi H., Liu X. (2021). An assessment of the effectiveness of China’s nature reservesfor mitigating anthropogenic pressures based on propensityscore matching. Acta Geogr. Sin..

[42] Li J.C., Wang W.L., Hu G.Y., Wei Z.H. (2010). Changes in ecosystem service values in Zoige Plateau, China. Agric. Ecosyst. Environ..

[43] Voll F., Luthe T. (2014). A systemic perspective on sustainable governance of protected areas. Eco. Mont..

[44] Wang Y.J., Yang H.B., Qi D.W., Songer M., Bai W.K., Zhou C.Q., Zhang J.D., Huang Q.Y. (2021). Efficacy and management challenges of the zoning designations of China’s national parks. Biol. Conserv..

[45] Kang D.W., Li J.Q. (2018). Role of nature reserves in giant panda protection. Environ. Sci. Pollut. Res..

[46] Wei W., Swaisgood R.R., Pilfold N.W., Owen M.A., Dai Q., Wei F.W., Han H., Yang Z.S., Yang X.Y., Gu X.D. (2020). Assessing the Effectiveness of China’s Panda Protection System. Curr. Biol..

[47] Jakes A.F., Jones P.F., Paige L.C., Seidler R.G., Huijser M.P. (2018). A fence runs through it: A call for greater attention to the influence of fences on wildlife and ecosystems. Biol. Conserv..

[48] Song Z.J., Zhou W., Gao L. (2021). Development of Giant Panda Nature Reserves in China: Achievements and Problems. J. For. Econ..

[49] Cameron R.P. (2006). Protected area—Working forest interface: Ecological concerns for protected areas management in Canada. Nat. Area J..

[50] Imhof R., Vogel M., Ruiz G. (2009). Mobility and Protected Areas in the Alps. Eco. Mont..

[51] Zeng F.W. (2014). An evaluation of residents’ perceptions of the creation of a geopark: A case study on the geopark in Mt. Huaying Grand Canyon, Sichuan Province, China. Environ. Earth Sci..

[52] Allendorf T.D., Yang J. (2013). The role of ecosystem services in park-people relationships: The case of Gaoligongshan Nature Reserve in southwest China. Biol. Conserv..

[53] Liu Y.M., Yang S.N., Han C.L., Ni W., Zhu Y.Y. (2020). Variability in Regional Ecological Vulnerability: A Case Study of Sichuan Province, China. Int. J. Disast. Risk Sci..

[54] Ruiz D., Moreno H.A., Gutierrez M.E., Zapata P.A. (2008). Changing climate and endangered high mountain ecosystems in Colombia. Sci. Total Environ..

[55] Chen L., Fan M., Wang Q. (2019). Spatial priority conservation areas for vegetation habitat across the Upper Reaches of Min River located in Sichuan Province, China. Glob. Ecol. Conserv..

[56] Miller-Rushing A.J., Primack R.B., Ma K.P., Zhou Z.Q. (2017). A Chinese approach to protected areas: A case study comparison with the United States. Biol. Conserv..

[57] Duniway M.C., Geiger E.L., Minnick T.J., Phillips S.L., Belnap J. (2018). Insights from Long-Term Ungrazed and GrazedWatersheds in a Salt Desert Colorado Plateau Ecosystem. Rangel. Ecol. Manag..

[58] Cloern J.E., Safran S.M., Vaughn L.S., Robinson A., Whipple A.A., Boyer K.E., Drexler J.Z., Naiman R.J., Pinckney J.L., Howe E.R. (2021). On the human appropriation of wetland primary production. Sci. Total Environ..

[59] Wu R.D., Hua C.L., Yu G.Z., Ma J.Z., Yang F.L., Wang J.J., Jin T., Long Y.C., Guo Y., Zhao H.W. (2020). Assessing protected area overlaps and performance to attain China’s new national park system. Biol. Conserv..

[60] Ye X., Liu G.H., Li Z.S., Wang H., Zeng Y. (2015). Assessing Local and Surrounding Threats to the Protected Area Network in a Biodiversity Hotspot: The Hengduan Mountains of Southwest China. PLoS ONE.

[61] Lu C.Y., Wang Z.M., Li L., Wu P.Z., Mao D.H., Jia M.M., Dong Z.Y. (2016). Assessing the conservation effectiveness of wetland protected areas in Northeast China. Wetl. Ecol. Manag..

[62] Kirkman S.P., Mann B.Q., Sink K.J., Adams R., Livingstone T.C., Mann-Lang J.B., Pfaff M.C., Samaai T., van der Bank M.G., Williams L. (2021). Evaluating the evidence for ecological effectiveness of South Africa’s marine protected areas. Afr. J. Mar. Sci..

[63] Belkayali N., Guloglu Y., Sevik H. (2016). What affects perceptions of local residents toward protected areas? A case study from Kure Mountains National Park, Turkey. Int. J. Sustain. Dev. World.

[64] Zhang H.Y., Fan J.W., Cao W., Zhong H.P., Harris W., Gong G.L., Zhang Y.X. (2018). Changes in multiple ecosystem services between 2000 and 2013 and their driving factors in the Grazing Withdrawal Program, China. Ecol. Eng..

