# Supplementary material for: Assessment of the Ecological Protection Effectiveness of Protected Areas Using Propensity Score Matching: A Case Study in Sichuan, China
Source: Int J Environ Res Public Health. 2022 Apr 18;19(8):4920. doi: 10.3390/ijerph19084920 (PMC9033088; doi:10.3390/ijerph19084920)
Supplement: Supplementary file 1 [file ijerph-19-04920-s001.zip › ijerph-1628998-supplementary.pdf]

Supplementary S1

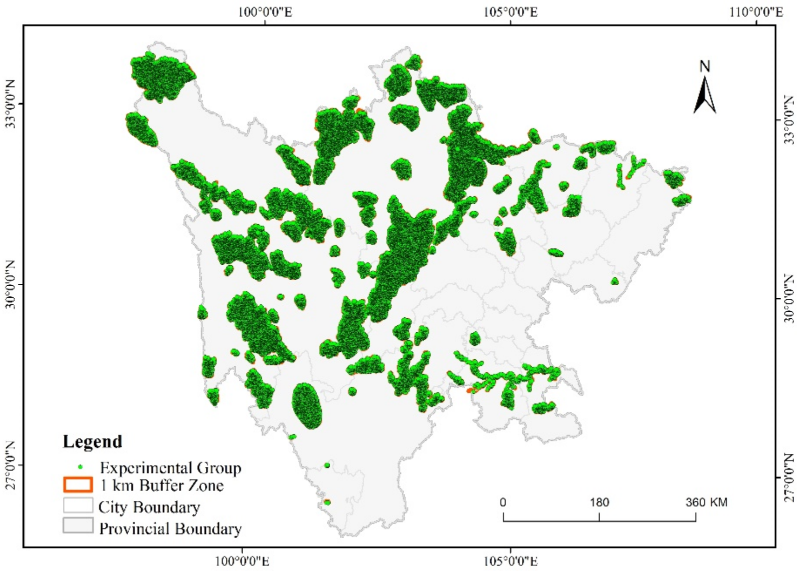

Figure S1. Points of the experimental group.

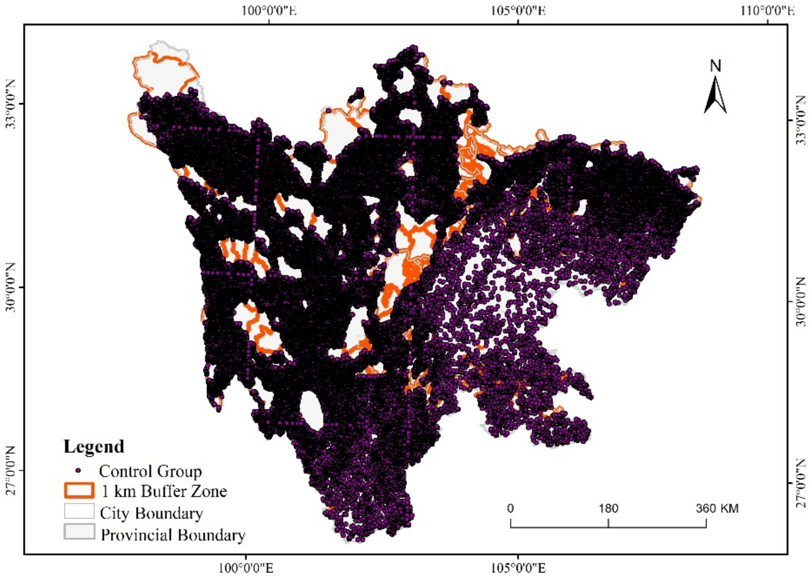

Figure S2. Points of the control group.

After we operated in accordance with the technical route,51,767 and 199,373 points inside and outside the protected areas were collected, respectively. The 9 influencing factors (e.g., elevation, etc.) were spatially joined with the points using the "Extract Multi Values to Points" tool. After completing the PSM analysis in Stata, the final matching results were obtained, the remaining 28,262 points in the experimental group and 63,936 points in the control group were used as matching points (Figure S1 and Figure S2).

Supplementary S2

Through the Stata software platform, we performed the common support test and the balance test on the matching results to validate the matching accuracy.

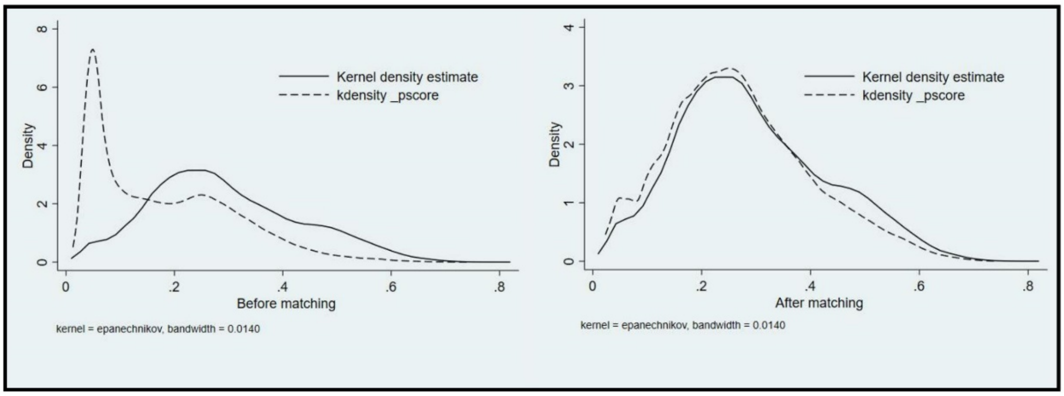

Figure S3. Nuclear density comparison chart.

The nuclear density map is the most commonly used type in the common support testing method, showing the fitting of the P-Score density distribution of the experimental group and the control group before and after matching. Figure S3 showed that after the matching, the two lines were very similar, that was, the range of the common support set increased, meanwhile, the difference between the experimental group and the control group decreased, too.

Table S1. Results of balance test.

Sample	Ps-R^2	LR chi^2	P>chi^2	Mean Bias	Med Bias	B
Unmatched	0.12	31091.78	0.00	46.90	42.70	93.00
Matched	0.01	144.50	0.71	1.90	1.90	7.40

In the balance test results (Table S1), the Ps-R^2 significantly reduced, and directly dropped to 0.01 after matching. The LR chi^2 dropped from 31091.78 before matching to144.50. Besides, the P value increased after matching, the mean bias and the med bias both reduced to 1.90%. Furthermore, the value of B was reduced from 93.00% to below 7.40% during the matching process. It revealed that the matching results were fully balanced, and the matching methods and rules were more rationally applied.

Supplementary S3

Table S2. List of the Types of Changes in Protection Effectiveness of Protected Areas in Sichuan Province.

type \ level	National	Provincial	County	Others
Effective	★(1)Sutie of Panzhihua<br>✷(5)Liziping, Huae Mountain, Longxi-Hongkou, Micang Mountain, White River<br>●(21)Meigudafengding, Laojun Mountain, Mabiandafengding, Qianfo Mountain, Xiaozhaizi Valley, Fengtongzhai, Wolong, Xuebaoding, Tangjia River, Wanglang, Jiuzhaigou, Rare aquatic animals in Nuoshui River Protected Area, Hongyagan Dam Giant PandaProtected Area, Yingjing Paocaowan Giant Panda Protected Area, Yingjing Sanhe Giant Panda Protected Area, Lushan Dachuan Giant Panda Protected Area, Dayi Yunhua Mountain Giant Panda Protected Area, Hongbaichang Giant Panda Protected Area, Xi River of Baoxing Giant Panda Protected Area, Taian River of Dujiangyan Giant Panda Protected Area, Hejia Mountain Giant Panda Protected Area,	★(1)Daxiaolangou<br>✷(3)Xiayong, Miyaluo, Xiaohegou<br>●(19)Lake Leiboma Giant Panda Protected Area, Mamize, Ma'an Mountain, Shenguozhuang, Zhile, Wawu mountain, Laba River, Mosika, Caopo Town, Jiuding Mountain, Baodinggou, Piankou, Baiyang, Heishui River,Anzi River, Dongyanggou, Huanglong, Maozhai, Wujiao	★(1)Malkang Minjiang Cypress Protected Area<br>✷(4)Huangjing, Erlang Mountain, Tianquan Erlang Mountain, Zhuchanghou<br>● (3) Baili Canyon, Daxiaogou, Longdishui<br>▲(1)Ruoergai Seat<br>✻ (1) Lingmen Mountain	★(1) Zemulong<br>✷(4)Matou Mountain, Jueluohuo, Emei Mountain, Zheduo Mountain<br>●(15)Zuantianpo, Leibo Leimaping Giant Panda Protected Area, Wuzhi Mountain of Pingshan, Ganluoxiluo Giant Panda Protected Area, Xianjiapu of Mabian Giant Panda Protected Area, Daqiao, Mianning Shangluba Giant Panda Protected Area, Jinkouhe Dawa Mountain Giant Panda Protected Area, Shimiancaoke Giant Panda Protected Area, Kangding Pengta Giant Panda Protected Area, Huangshui River, Weimengou of Mao Country, Qingpian River of Beichuan Country, Baicao River, Pingwu Country Protected Area,<br>▲(2)Daqiao Reservoir Protected Area, Wenchuan Sanjiang Ecological Tourism Zone
Ineffective	✷(3)Changning Bamboo Sea, Aden, Gongga Mountain,<br>●(4)Rare Endemic Fish in the Upper Reaches of the Yangtze River Protected Area, Xiaojinsiguniang Mountain, Changshagongma, Chaqingsongduo White-lipped Deer Protected Area<br>▲(3)Haizi Mountain, Nanmoge Wetland, Ruoergai Wetland	★(1)Cuiyunlang Gubai Protected Area<br>●(6)Hongba, Wahui Mountain, Zhubalong,Taining Yuke, Xinlu Sea, Luoxu<br>▲(2)Kasha Lake, Lugu Lake<br>□(1)Suishui sponge reef Protected Area	★(1) Daoxugou<br>✷(4)Niding Grand Canyon, Arengou, Duopugou, Dugoula<br>●(18)Gajin, Fozhu Canyon, Suochong, Genieshen Mountain, Taizhangou, Cuopugou, Kasongdu, Yirigou, Ribaxue Mountain, Amula, Lengdagou, Kaniang Town, Lang Village,Mailajiangcuo, Xionglongxi, Keluodong, Nianlong, Yanboyeze Mountain<br>▲(8)Gunba, Zagashen Mountain, Gemu, Mianyang Egret Forest Protected Area, Gouxi River Wetland, Youyi, Riganqiao, Luhu White Stork Grey Crane Protected Area	●(1) Taihe Egret Protected Area,<br>▲(1) Wenxixi River

type \ level	National	Provincial	County	Others
Fluctuating	✶(1) Huagao Creek<br>✷(1) Baishui River<br>●(1)Gexigou	✶(1)Alsophila spinulosa Protected Area<br>✷(1)Guanwu Mountain<br>●(4)Jintang Kongyu, Sandagu, Shuimogou,Tiebu<br>▲(1)Manzetang Wetland	✷(1) Jiulong Mountain<br>●(2)Kowloon Millennium Snow Giant Panda Protected Area,<br>Sichuan Little Golden Monkey Protected Area	✷(1) Gucheng Mountain

Note: The types represented by different symbols are: ✶Wild Plant ✷Forest Ecology ●Wild Animal ▲Wetland Ecology □Paleontological Remains ❇ Geological Heritage, a total of 149 protected areas.
